# *Enterococcus faecalis*-associated lung abscess in a male adolescent- a case report

**DOI:** 10.1186/s12887-020-2003-8

**Published:** 2020-03-02

**Authors:** Ana Raquel Mendes, António Costa, Helena Ferreira, Cristina Ferreira

**Affiliations:** 1 Pediatric Department, Centro Materno-Infantil do Norte, Centro Hospitalar e Universitário do Porto, Largo da Maternidade de Júlio Dinis, 4050-651 Porto, Portugal; 2 Pneumology Department, Hospital da Senhora da Oliveira, Rua dos Cutileiros 114, Creixomil, Guimarães, Portugal; 3 Pediatric Department, Hospital da Senhora da Oliveira, Rua dos Cutileiros 114, Creixomil, Guimarães, Portugal

**Keywords:** Adolescent, *Enterococcus faecalis*, Lung abscess

## Abstract

**Background:**

Enterococci are rarely considered pulmonary pathogens; they are usually regarded as colonizers of the airway. The authors present the case of a previously healthy male adolescent, with complaints of fatigue and chest pain, who was diagnosed with *Enterococcus faecalis*-associated acute primary lung abscess.

**Case presentation:**

A previously healthy 17-year old boy was admitted to the pediatric ward due to a one-week history of fatigue, inspiratory left side chest pain, dry cough and nasal obstruction. On admission at the emergency department, he was afebrile, with no signs of respiratory distress, but with diminished breath sounds on the left side. A chest x-ray showed a round opacity on the posterior basal segment of the left lower lobe; he was discharged with oral amoxicillin 1000 mg three times a day with the diagnosis of community-acquired pneumonia. Due to the worsening of the productive cough with purulent stinking sputum he was re-evaluated after 4 days. Laboratory studies showed a leukocyte count of 15200/uL and a c-reactive protein of 172 mg/l. The chest computed tomography scan was suggestive of a consolidation of the left lower lobe base and a central abscess. An intravenous course of ceftriaxone and clindamycin was initiated, with a favourable clinical evolution. The bronchofibroscopy performed on day four after his admission revealed the presence of a tracheal bronchus and numerous purulent secretions. Culture examination of bronchoalveolar lavage fluid samples was positive (> 10^5) for *Enterococcus faecalis*. No complications were registered during his stay in the pediatric ward. He was discharged after a 14-day course of intravenous ceftriaxone and clindamycin, with the recommendation to complete a four-week course of oral amoxicillin/clavulanic acid. On his reevaluation 4 weeks after his discharge, he was asymptomatic.

**Conclusion:**

This case report highlights the importance of considering *Enterococcus faecalis* as an etiologic agent in cases of non-resolving or complicated cases of pneumonia, such as lung abscesses, even in young patients with no comorbidities or risk factors.

## Background

Community-acquired pneumonia (CAP) is one of the most common diagnosis in pediatric age, with an incidence of approximately 14.4 per 10,000 cases in children aged more than 5 years and 33.8 per 10,000 cases in children under 5 years in Europe [1]. Complications of bacterial pneumonia include pneumatoceles, pleural effusion, empyema, necrotizing pneumonia and lung abscesses. Lung abscess is an uncommon condition in children, with an estimated incidence of 0.7 per 100,000 annual cases [2], being the most frequently involved organisms the anaerobic flora of the upper respiratory tract and *S. aureus* [3].

Enterococci are part of the commensal flora on human gut surface and airways; in some cases, they can be isolated from the vagina and the oral cavity. It is generally considered to be a rare cause of respiratory illnesses; nevertheless, it may be responsible for sinusitis, infections of the trachea and bronchi, pneumonia, lung abscesses and pleural empyema [4]. The authors present the case of a previously healthy male adolescent, with complaints of fatigue and chest pain, who was diagnosed with *Enterococcus faecalis*-associated acute primary lung abscess in the course of the investigation.

## Case presentation

A previously healthy 17-year old boy was admitted to the pediatric ward due to a one-week history of fatigue, inspiratory left side chest pain with the intensity of 7/10, dry cough and nasal obstruction. He did not report any recent history of fever, dyspnea, night sweats, hemoptysis, ear or throat pain, abdominal pain, weight loss or recent alterations in his bladder and bowel habits. There were no known recent contacts with people presenting with infectious diseases, including tuberculosis. He recalled having had a fall and having landed on his left side while playing football 1 week prior to the onset of the symptoms. His family medical history and his past personal medical background was unremarkable except for an episode of acute bronchiolitis and an episode of community-acquired pneumonia that required hospitalization at the age of 2 years. He maintained a regular follow-up in the immunoallergology outpatient clinic due to a history of allergic rhinitis. His vaccine schedule was updated according to the national vaccination programme.

The adolescent denied alcohol or smoking habits and recreational drug use.

**Fig. 1:**
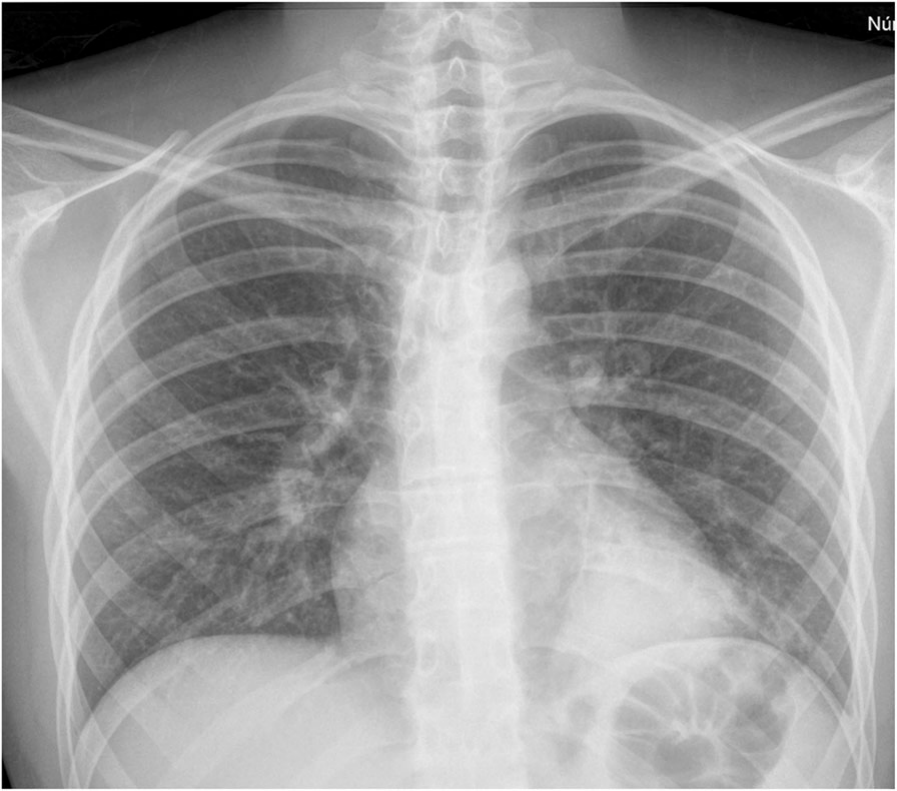
Chest x-ray performed on his first emergency department visit

On admission at his first visit to the emergency department, he was afebrile, with no signs of respiratory distress, but with diminished breath sounds on the left side. He complained of pain on palpation of the left lower costal margin. A left costal grid and a chest x-ray was performed (Fig. 1), which showed the same opacity on the posterior basal segment of the left lower lobe and no signs of rib fracture; thus, the patient was discharged with oral amoxicillin 1000 mg three times a day with the diagnosis of community-acquired pneumonia.

**Fig. 2:**
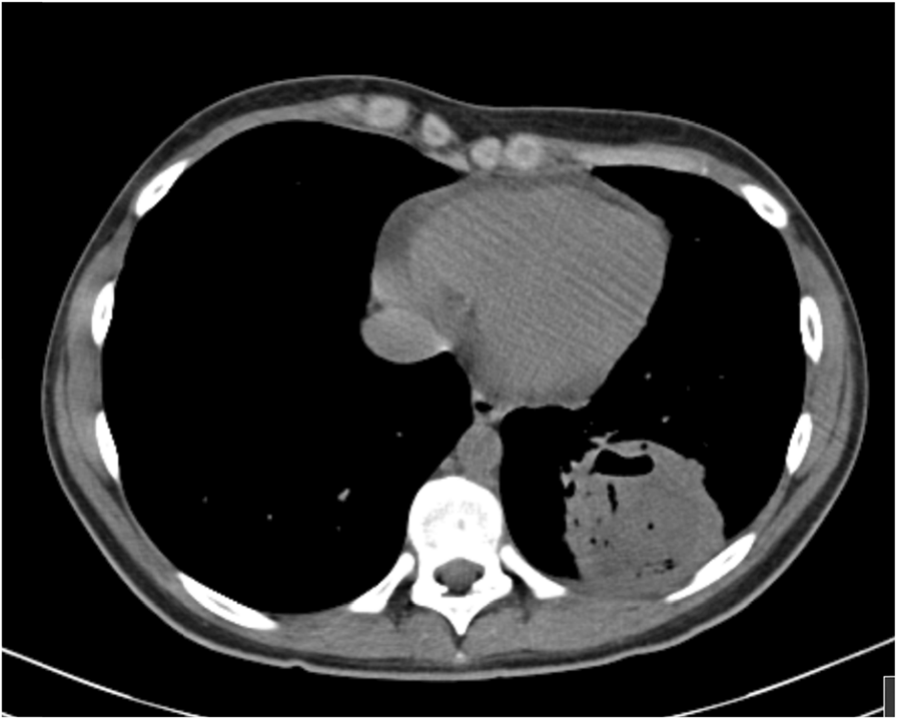
Transverse plane of the chest CT scan showing the presence of the abscess

**Fig. 3:**
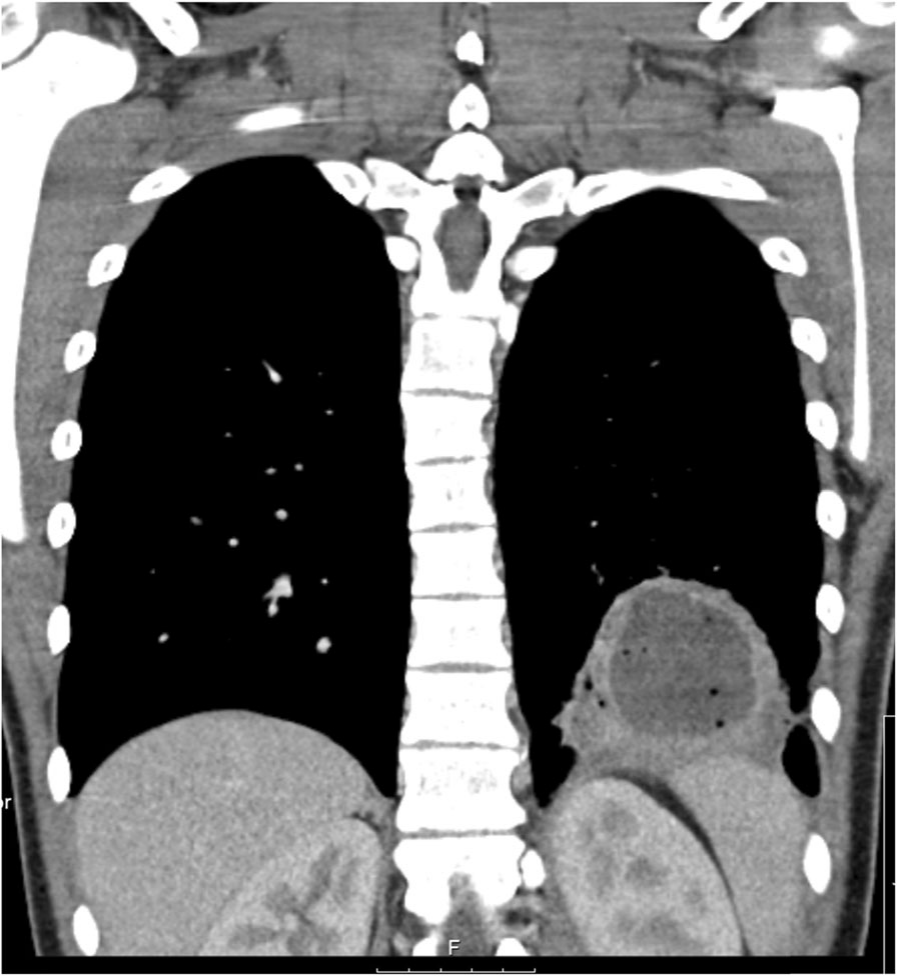
Frontal plane of the chest CT scan

Due to the progressive worsening of the productive cough with emission of purulent stinking thick green sputum, he returned to the emergency department 4 days after. On admission, he had a peripheral oxygen saturation of 98% (FiO2 21%), a heart rate of 95 bpm and an axillary temperature of 36.7 °C. His physical appearance was described as sickly, with no signs of respiratory distress, but with diminished breath sounds on the left lower lung, barely audible on the bases, and with crackles on the inferior two thirds of the left lung. The chest x-ray showed the same retrocardiac opacity on the left lower lobe. Laboratory studies including a complete blood count, glucose, ionogram, renal and hepatic function and c-reactive protein were normal, except for a leucocyte count of 15200/uL (neutrophil count of 12500/uL and lymphocyte count of 1700/uL) and a c-reactive protein of 172 mg/L. A chest computed tomography (CT) scan was ordered, which was suggestive of a consolidation with air bronchogram of the left lower lobe base associated with necrotizing characteristics and a central abscess measuring 5 cm in its greatest diameter (Figs. 2 and 3). Immunoglobulin, complement and alpha-1-antitrypsin levels were normal. Viral markers for hepatitis B and C and Human Immunodeficiency Virus (HIV) were negative. Serologic markers for rubeola, toxoplasmosis, CMV, EBV, herpes simplex virus, adenovirus, *Chlamydia trachomatis* and *Chlamydia pneumoniae, Brucella and Mycoplasma pneumoniae* showed no evidence of active infection. The virologic exam of nasopharyngeal aspirate and the bacteriologic and mycobacteriologic exam of the sputum were negative. Blood cultures for aerobic and anaerobic agents were negative.

It was decided to admit the patient to the pediatric ward in order to administer intravenous antibiotics and to maintain a closer surveillance.

A course of intravenous ceftriaxone 2 g twice a day and clindamycin 30 mg/kg/day twice a day was initiated, with a favourable clinical outcome. He ceased complaining of chest pain on day three and reported a significant improvement of his cough. A single episode of fever was recorded on day seven without any further complications. After a consult with pneumology, it was decided to submit the patient to a bronchofibroscopy on day four after his admission. As his coagulation studies were abnormal with a prothrombin time (PT) of 16.7 s and a ratio of 1.5, 10 mg of intravenous vitamin K was administered twice with a decrease to a PT value of 15.9 s and a ratio of 1.4. A mixing test was ordered, with the result of a probable coagulation factor deficiency.

The bronchofibroscopy revealed the presence of a tracheal bronchus as well as numerous purulent secretions in the left posterior basal segmental bronchus, and excluded bronchial obstruction. Culture examination of bronchoalveolar lavage (BAL) fluid samples was positive (> 10^5) for *Enterococcus faecalis* (*E. faecalis*), which was sensitive to ampicillin, imipenem, nitrofurantoin, linezolid, teicoplanin, vancomycin and tigecycline, and resistant to levofloxacin, quinupristin/dalfopristin, and trimethoprim/sulfamethoxazole. BAL was negative for *Mycobacterium tuberculosis* DNA. Thus, it was decided to complete a 14-day course of intravenous antibiotics with ceftriaxone and clindamycin. An abdominal ultrasound was performed before he was discharged, which excluded the presence of a thoraco-abdominal fistula.

**Fig. 4:**
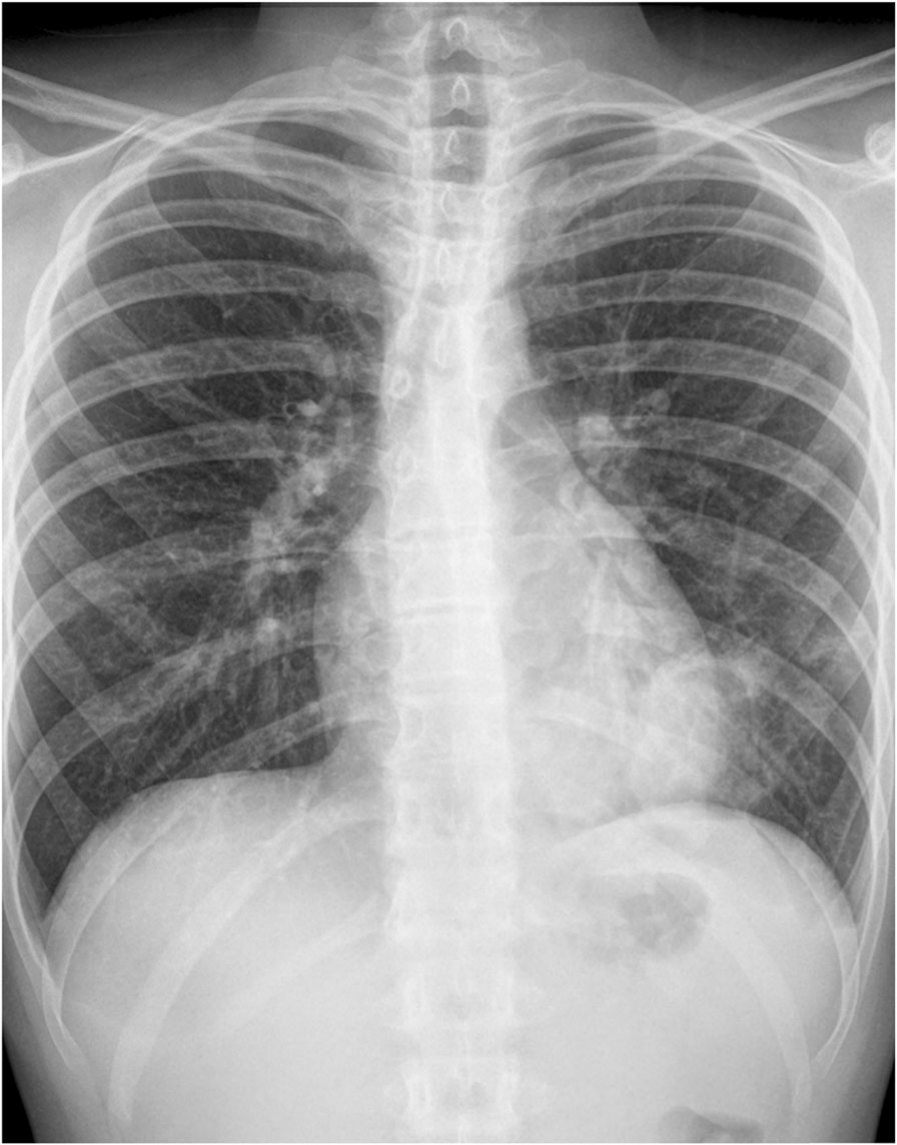
Frontal chest x-ray performed on day 14 showing an improvement of the opacity

**Fig. 5:**
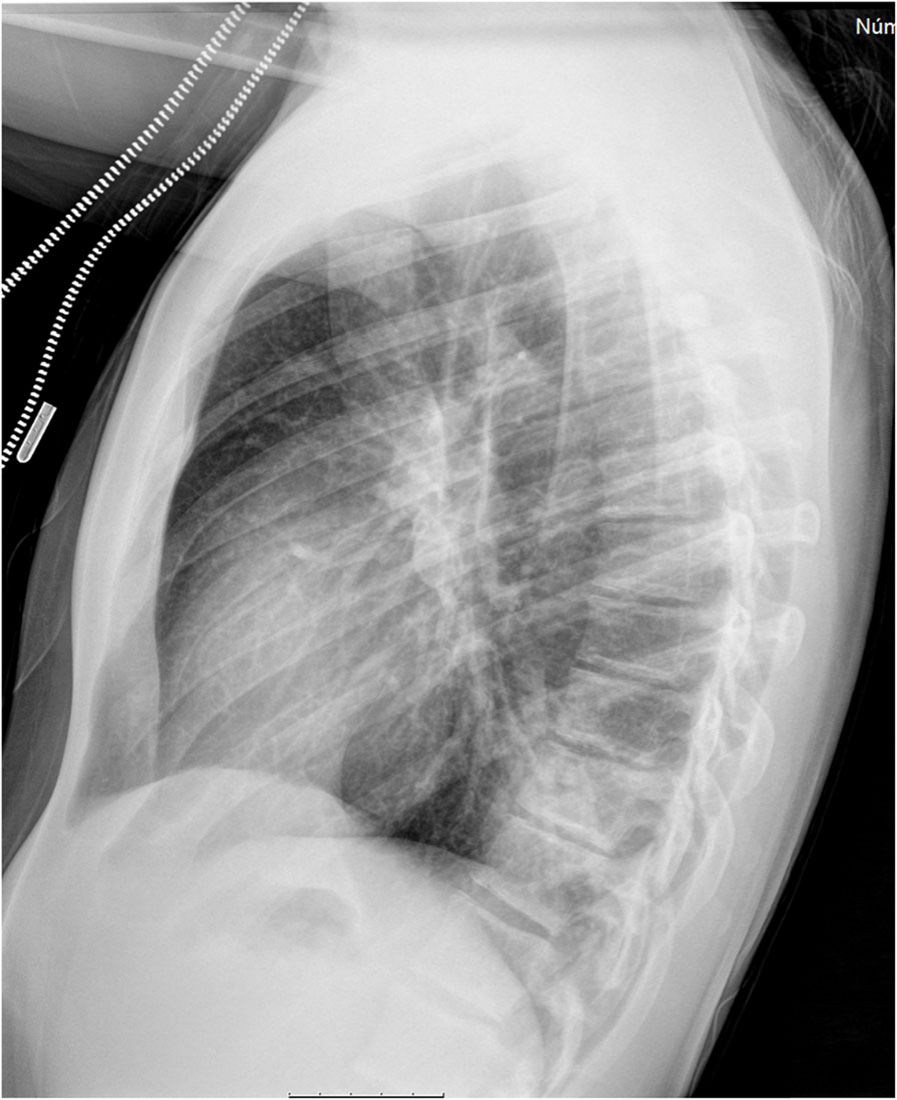
Left lateral chest x-ray performed on day 14 showing an improvement of the opacity

No complications were documented during his stay in the pediatric ward. A chest x-ray was repeated on day 12 showing an improvement of the opacity (Figs. 4 and 5).

He was discharged after 14 days with the recommendation to complete a four-week course of oral amoxicillin/clavulanic acid 875 mg/125 mg three times a day. He was referred to the pediatric and to the hematology outpatient clinic.

On his reevaluation 4 weeks after his discharge, he denied dyspnea, cough, chest pain, hemoptysis, respiratory distress or anorexia. His physical exam was unremarkable except for some crackles on the left lower lobe base and a slight decrease of breath sounds on the same side. The sweat test was negative. The patient completed the course of oral antibiotics without any complications; he is currently asymptomatic.

## Discussion and conclusion

Enterococci are rarely considered pulmonary pathogens; when isolated from respiratory samples, they are usually considered colonizers of the airway [5].

However, the paucity of reports may reflect the erroneous dismission of isolates as contaminants [4]. Furthermore, the indolent disease course and the initial improvement without an appropriate antibiotic therapy supports the hypothesis of under-diagnosis [6]. Additionally, the frequently used empiric treatments for pneumonia, which contain penicillin or amoxicillin may resolve most of the lower respiratory airway diseases, contributing to a low number of case reports [6].

Case reports of *Enterococcus* spp. pneumonia, empyema and lung abscesses have been described, especially in patients with underlying diseases, including cirrhosis, alcoholism and nephrotic syndrome [5]. A study of serious infections due to *Enterococcus* concluded that over the course of 1 year 110 enterococcal infections were found across six hospitals, with 4% of those infections located in the respiratory tract [7].

One of the first case reports of enterococcal lung abscess was described as early as 1974 [8]. Most of the cases reported in literature occurred in elderly patients with co-morbidities (stroke, hypertension, vascular disease, kidney transplant, HIV infection) and/or risk factors (aspiration, alcoholism, tobacco) [4, 5].

As far as we know, this is the first report of *E. faecalis* primary lung abscess in an adolescent. In contrast to most cases that have been described, our patient did not have any previously known co-morbidities nor did the investigation reveal any of the aforementioned risk factors.

Clinical presentation is variable, as it ranges from high fever, productive cough and pleuritic chest pain [6]. Our patient presented with all of these features except for high fever. In fact, during the course of his disease and its treatment, only one episode of fever was recorded. In the series described by Grupper M et al., patients were also reported to have leukocytosis and/or left shift, abnormal pulmonary auscultatory findings and pulmonary lobar infiltrates [6], which also was found in our patient. Thus, symptoms, signs, laboratory and image findings of *Enterococcus*-associated lower respiratory tract infections resemble those of a typical bacterial pneumonia [6].

*E. faecalis* was isolated from BAL samples obtained in our patient by bronchofibroscopy, so one could consider the isolated agent as contaminant or colonizer. Nevertheless, isolation of *Enterococcus spp.* from respiratory samples also varies according to the literature- it ranges from bronchial aspirate obtained by fiberoptic bronchoscope, transtracheal sputum and bronchial secretions obtained by bronchoscopy [7], lung tissue obtained by lung needle biopsy [5], to BAL samples [9]. In our case report, *E. faecalis* was isolated as a sole pathogen.

The currently recommended empiric regimen for community-acquired pneumonia does not cover enterococcal infection [6]. The most adequate treatment for *Enterococcus*-associated airway disease has to be defined case-by-case; a combination of antibiotics and empyema/ abscess drainage may be required [4]. Various antibiotics regimens and combinations have been used as reported in the literature, such as penicillin, amoxicillin/clavulanate and ampicillin, fluoroquinolones, gentamicin, vancomycin, aeromycin and streptomycin and linezolid [5, 6]. Thus, no conclusions can be drawn with regards to the most appropriate treatment regimen. Most authors seem to agree that the antibiotics choice should be based on susceptibility testing [5]. Our patient showed a favourable evolution with a 14-day course of intravenous ceftriaxone+clindamycin, combined with a four-week course of oral amoxicillin/clavulanate. No surgical intervention for resolution was required.

Obtaining appropriate cultures in all non-resolving community cases of pneumonia is paramount, especially because the non-specific clinical picture of *Enterococcus*-associated lung abscess may delay the initiation of an adequate treatment [6]. This was illustrated by our case report, where all culture exams were negative, except for the BAL samples.

Regarding the outcome, in the case series reported by Grupper M et al., complications were described in six out of nine patients, and two of them died during hospitalization [6]. In contrast, Vanschooneveld T et al. describe 11 cases of *Enterococcus* pleuro-pulmonary infections, where clinical cure was achieved in all except for one patient, where data was not available. Therefore, patients with *Enterococcus* pleuro-pulmonary infections appear to have good clinical outcomes [5]. This is in accordance with our case report as our patient obtained an apparent clinical cure with intravenous and oral antibiotics treatment. His young age and absence of co-morbidities or risk factors may have contributed to the favourable clinical outcome.

We conclude that, although Enterococcal-associated lower respiratory tract infections are infrequently reported, it is important to keep this pathogen in mind in non-resolving, severe or complicated cases of pneumonia, even in young patients in the absence of comorbidities or risk factors.

## Data Availability

All data analysed during this study are included in this published article and its supplementary information files.

## Abbreviations

BAL: Bronchoalveolar lavage
CAP: Community-acquired pneumonia
CT: Computed tomography
*E. faecalis*: *Enterococcus faecalis*
HIV: Human Immunodeficiency Virus
PT: Prothrombin time
